# Long-Term Safety of Intraperitoneal Radio Transmitter Implants in Brown Bears (*Ursus arctos*)

**DOI:** 10.3389/fvets.2018.00252

**Published:** 2018-10-15

**Authors:** Jon M. Arnemo, Bjørnar Ytrehus, Knut Madslien, Jonas Malmsten, Sven Brunberg, Peter Segerström, Alina L. Evans, Jon E. Swenson

**Affiliations:** ^1^Department of Forestry and Wildlife Management, Inland Norway University of Applied Sciences, Koppang, Norway; ^2^Department of Wildlife, Fish and Environmental Studies, Swedish University of Agricultural Sciences, Umeå, Sweden; ^3^Norwegian Institute for Nature Research, Trondheim, Norway; ^4^Norwegian Veterinary Institute, Oslo, Norway; ^5^Department of Pathology and Wildlife Diseases, National Veterinary Institute, Uppsala, Sweden; ^6^Scandinavian Brown Bear Research Project, Orsa, Sweden; ^7^Department of Ecology and Natural Resources Management, Norwegian University of Life Sciences, Ås, Norway

**Keywords:** biocompatibility, brown bear, foreign body reaction, implant, intraperitoneal, long-term safety, transmitter, *Ursus arctos*

## Abstract

Intraperitoneal radio transmitters have been widely used in free-ranging wild mammals, but there are no long-term studies on their biocompatibility or technical stability within the abdominal cavity of animals. Possible negative health effects may bias results from ecological studies on instrumented animals and raise concerns over animal welfare issues. The aim of this study was to evaluate the long-term technical stability and pathological effects of Telonics intraperitoneal very high frequency (VHF) radio transmitters in brown bears (*Ursus arctos*). We instrumented 305 individual bears with intraperitoneal VHF radio transmitters during a 19-year period. We surgically removed devices that had been in bears for 1–9 years and collected transmitters from animals that died 1–13 years after implantation. We took biopsies for histopathology from tissue encapsulating implants in live bears. Retrieved transmitters underwent a technical inspection. Of the 125 transmitters removed from live bears, 66 were free-floating in the peritoneal cavity [a mean (SD) of 3.8 (1.5) years after implantation], whereas 59 were encapsulated in the greater omentum [4.0 (1.8) years after implantation]. Histopathology of biopsies of the 1–15 mm thick capsules in 33 individuals showed that it consisted of organized layers of connective tissue. In one third of the bears, the inner part of the capsule was characterized by a foreign body reaction. We inspected 68 implants that had been in bears for 3.9 (2.4) years. The batteries had short-circuited four (5.9%) of these devices. This resulted in the death of two animals 10 and 13 years after implantation. In two other bears that underwent surgery, we found the short-circuited devices to be fully encapsulated within the peritoneal cavity 5 and 6 years after implantation. A significant proportion of the other 64 inspected implants showed serious technical problems, such as corrosion of metal parts or the batteries (50%), detachment of the end cap (11.8%), and erosion (7.4%) or melting (5.9%) of the wax coating. We conclude that the wax coating of the transmitters was not biocompatible, that the technical quality of the devices was poor, and that these implants should not be used in brown bears.

## Introduction

Implanted devices used in human medicine must provide science-based evidence of both the functional performance of the device and its compatibility and stability within the body of an animal before they can be approved for routine application in humans (1). There are no such requirements for implanted devices used in wildlife research. Cattet (2) reviewed the websites of six radio telemetry manufacturers in North America and found that none of them provided science-based evidence of the compatibility and stability of their products within the body cavity of an animal. Instead, the focus was on the functional performance of the device, such as battery-life and transmission range.

The use of implantable telemetry in animals started in the 1950s. Early reports focused on technical aspects of the implanted devices (3). The first reports on the use of abdominal radio telemetry in free-ranging mammals appeared in the 1970s (4, 5). In collaboration with Telonics Inc. (Mesa, AZ, USA), Melquist and Hornocker (6) developed intraperitoneal radio transmitters for use in North American river otters (*Lontra canadensis*). Since then, Telonics and other companies have been marketing intraperitoneal radio transmitters for a wide range of wildlife species.

We reviewed more than 1,500 publications on the use of implantable radio transmitters and other devices in wild mammalian species, ranging in body size from 4 g suckling white-footed mice (*Peromyscus leucopus*) (7) to adult grizzly bears (*Ursus arctos*) (8). We could not find any published studies on the long-term technical stability or biocompatibility of implanted radio transmitters and we identified only one peer-reviewed paper with a large sample size and a long time-span on health effects of such devices: Van Vuren (9) carried out 300 surgeries on 183 individual yellow-bellied marmots (*Marmota flaviventris*) in order to implant or replace intraperitoneal radio transmitters. He followed implanted animals for up to 4 years and concluded that the implants did not affect survival, growth, or reproduction. The devices were, however, clearly not biocompatible because he reported that “surgery to replace transmitters often revealed a thick, fibrous, sometimes highly vascularized membrane encasing the transmitter.” Case reports and anecdotal observations indicate that implants may cause serious health problems, including mortalities, months to several years post-surgery (10–13).

Cattet (2) raised concerns over animal welfare issues and the lack of knowledge about implanted devices in wildlife. Reports on king penguins [*Aptenodytes patagonicus*; (14)], marine mammals (15), Burchell's zebras [*Equus burchelli antiquorum*; 16), European badgers [*Meles meles*; 17), and caribou [*Rangifer tarandus*; 18) documented the need for long-term investigations on possible impacts of instrumentation of wildlife. Here we present data from a 19-year study on the technical stability and pathological effects of intraperitoneal radio transmitters in free-ranging European brown bears (*Ursus arctos*).

## Materials and methods

The present work was part of an ongoing ecological study by the Scandinavian Brown Bear Research Project (SBBRP) (19). From 1997 to 2015, we carried out a total of 446 surgeries according to an established protocol (20) to implant, replace, or remove Telonics intraperitoneal very high frequency (VHF) radio transmitters involving 305 individual free-ranging brown bears [213 yearlings (162 females, 51 males), 44 subadults (2–4 years, 28 females, 16 males), 48 adults (≥5 years, 20 females 5–27 years, 18 males 5–22 years, age missing for 3 females and 3 males), the age refers to the time of first implant]. Fifteen bears that previously had their implants removed underwent a second surgery after 1–9 years to receive a new implant. We used the following models (number of units, length × diameter, weight): IMP/400/L (*n* = 238, 15.2 × 3.3 cm, 170 g), IMP/700 (*n* = 139, 15.2 × 3.3 cm, 158 g), IMP/400/L/HP (*n* = 4, 21.0 × 3.3 cm, 240 g), and IMP/400 (*n* = 7, 9.7 × 3.3 mm, 95 g).

**Figure 1:**
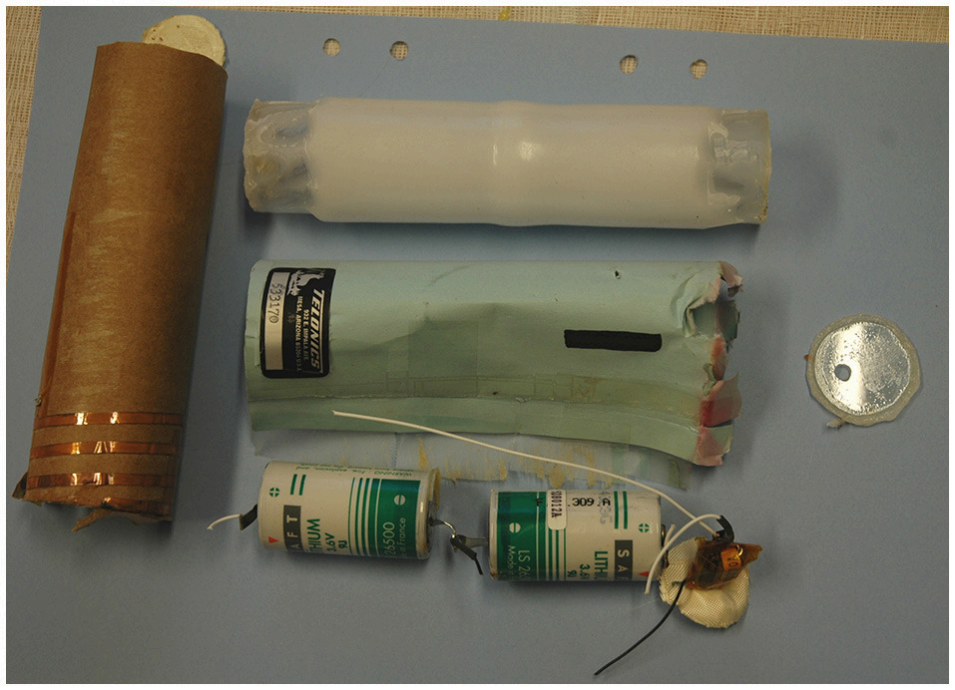
Components of a Telonics IMP/400/L intraperitoneal VHF implant (length 15.2 cm, diameter 3.3 cm, weight 170 g). The lithium batteries, transmitter, and antenna (central bottom) were enclosed in a thick paper tube (left), wrapped in thin paper (central middle) labeled with the company's name and address and the serial number of the device. These parts were contained within a plastic shell cylinder (central top) with both ends closed with glued-on end caps (right). The plastic shell cylinder was coated with a 2.1 mm thick wax of unknown composition.

The basic components of a Telonics IMP/400/L implant are shown in Figure 1. The lithium batteries, transmitter, and antenna were enclosed in a thick paper tube, wrapped in thin paper labeled with the company's name and address and the serial number of the device. These components were contained within a plastic shell cylinder with both ends closed with glued-on end caps. The plastic shell cylinder was coated with a 2.1 mm thick wax of unknown composition.

We inspected implants retrieved from live or dead bears for signs of discoloration, wear, or melting of the wax coating. The wax was then removed, the plastic shell cylinder was inspected for signs of fissures or cracks, and the attachment and sealing of the end caps were assessed. The end caps were removed and the internal parts were removed and inspected; the paper wrappings for dryness, the batteries for signs of short-circuiting and leakage, and all metal parts, including the batteries, for corrosion.

We collected biopsies from the tissue encapsulating the implants. We preserved the tissue samples in 10% phosphate-buffered, neutral formalin (Apotekproduksjon AS, Oslo, Norway) and shipped them to the Norwegian Veterinary Institute (Oslo, Norway) for histopathology. When we found pus-like content indicating possible bacterial growth inside the capsules, we used swabs (Swab-kit, Jan F. Andersen, Jevnaker, Norway) to collect samples, which were shipped with no cooling to the Norwegian Veterinary Institute for culturing by standard methods within 3 days. Thick tissue capsules (>2 mm) and capsules attached to a twisted loop of the omentum were amputated. We inspected and described biopsies before cutting 3–4 mm thick slices perpendicularly to the longitudinal axis of the capsule. The tissue slabs were dehydrated in ethanol, fixed in xylene, and embedded in paraffin before 5–6 μm thin sections were made, mounted, and stained with haemotoxylin-eosin and van Giessen according to standard procedures.

There were two major reasons to use implants in the bears. The SBBRP has a goal to follow individual bears throughout their lives and VHF implants allowed the recapture of individuals with neck collars that had been lost or had malfunctioned. The second reason was to avoid equipping yearling bears with transmitters mounted on neck collars, because young, growing bears would have to be recaptured annually for several years to change the collars. Capture and surgical protocols were approved by the Swedish Ethical Committee on Animal Research (Uppsala, Sweden; #C18/15), the Swedish Environmental Protection Agency (Stockholm, Sweden; NV-0758-14), and the Swedish Board of Agriculture (#31-11102/12).

## Results

At the time of denning in 2015, the 305 individual bears that had received implanted transmitters in our study had the following outcomes: for those still carrying a VHF implant [years refer to time after last implantation, given as mean (SD) (range)], 50 were alive 1.4 (±1.3) (0–5) years later, 39 were missing (no radio signals) 2.4 (±1.4) (0–6) years later, 129 had been shot (legally or illegally) 1.9 (±2.3) (0–13) years later, 20 had been killed by another bear 0.8 (±0.9) (0–3) years later, two died due to leakage from short-circuited batteries 10 and 13 years after surgery, two had died from trauma 2 months (hit by a train) and 2 years (crushed by a sliding rock) later, three had died during or shortly after capture (two from drowning and one due to dehiscence of the surgical wound), and 27 had died from unknown causes 1.3 (1.8) (0–8) years later (inconclusive necropsies, decomposed or partly eaten carcasses, implant found without any remains of the bear). Of those from which the VHF had been removed (time after removal of the implant), 17 were missing (removal, loss, or malfunctioning of radio collar) 3.1 (±2.4) (0–7) years later, 15 were shot 2.1 (±2.2) (0–8) years later, and one had been killed by another bear 1 year later.

**Figure 2:**
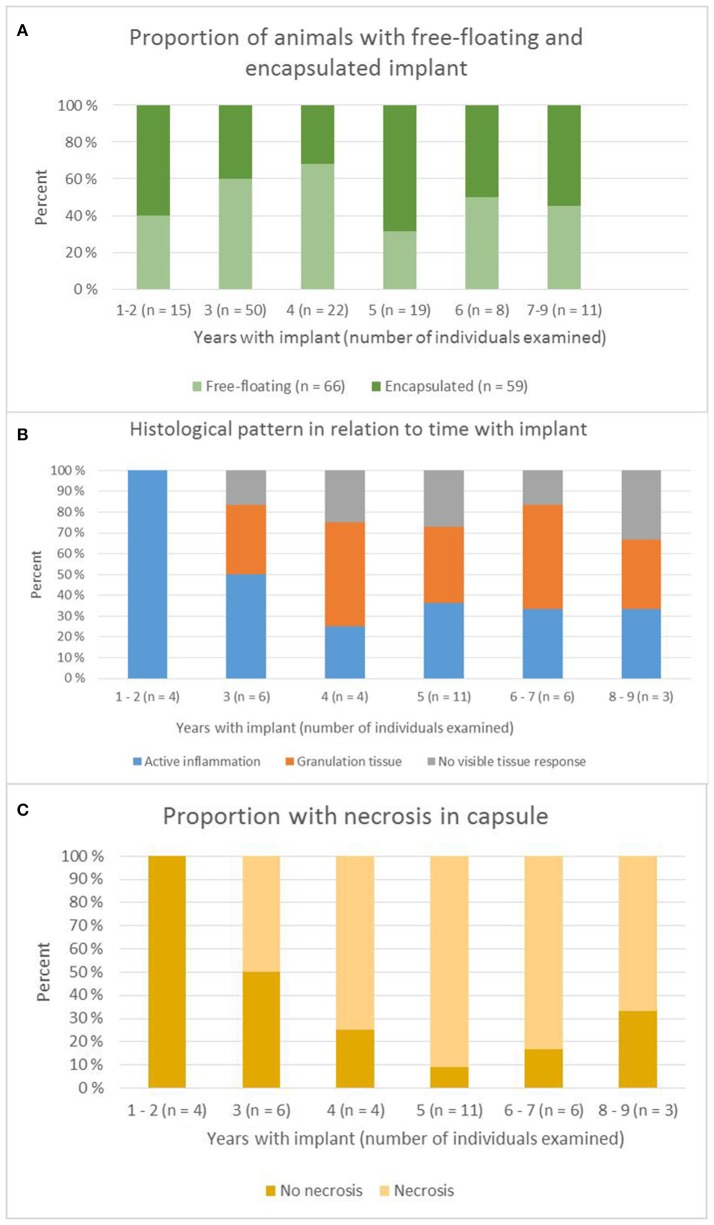
**(A)** Proportion of bears with free-floating and encapsulated implant in relation to years with the device. **(B)** Histological pattern in relation to years with implant. **(C)** Proportion of capsules with necrosis in relation to years with implant.

From 2000 to 2015, we conducted 125 surgeries to remove implants that had been carried for 1–9 years. In 66 (53%) of the bears, the implants were found free-floating with no encapsulation in the peritoneal cavity and could be easily removed. In 59 (47%) of the cases, the transmitters were found trapped in the greater omentum and encapsulated by 1–15 mm thick layers of connective tissue with various degrees of vascularization. In 23 of the bears (39% of those with encapsulated transmitters), we amputated the connective tissue capsule and parts of the omentum. The bears with free-floating implants had carried the implant for 3.8 (1.5) years and those with encapsulated devices for 4.0 (1.8) years. There was no significant difference in time between these two groups (Student's *t*-test, *p* = 0.42). Figure 2A shows the proportion of encapsulated devices over time.

Two animals (Cases 1 and 2 below) with encapsulated implants had aggregates of thick, opaque and yellowish exudate between the capsule and the implant. One of them (Case 2) also had a 5-cm wide cystic mass of tissue, containing similar exudate attached to the outer surface of the capsule. Bacteriological swabs from both cases were negative. An additional bear (W1211), an adult male whose implant was replaced after 3 years, had a cystic structure, associated with the capsule, filled with a similar exudate as described above, but the material was not cultured.

We inspected 68 implanted transmitters that had been in bears for 3.9 (2.4) (0–13) years. Of these, 54 transmitters were surgically removed from live bears and 14 were retrieved from dead bears (ten shot by hunters, two killed by the transmitter, and two killed by bears). All implants showed some degree of yellowish discoloring of the wax coating. In four (5.9%) of the implants, parts of the wax had partially melted and the underlying plastic shell cylinder was visible. In five (7.4%) of the implants, the wax was visibly thinner than on new transmitters. We interpreted this as erosion of the wax due to wear. In one (1.5%) of the implants, the plastic shell cylinder had a longitudinal crack. One of the end caps was loose or open in eight (11.8%) of the implants. One (1.5%) implant had visible moisture condensed on the inside of the plastic shell cylinder. In 32 (50.0%) of the 64 intact implants, moisture had resulted in corrosion of the batteries and other metal parts. Leakage from the batteries was seen in one (1.5%) otherwise intact implant. In four (5.9%) of the implants, the batteries had short-circuited (Cases 3–6 below).

**Figure 3:**
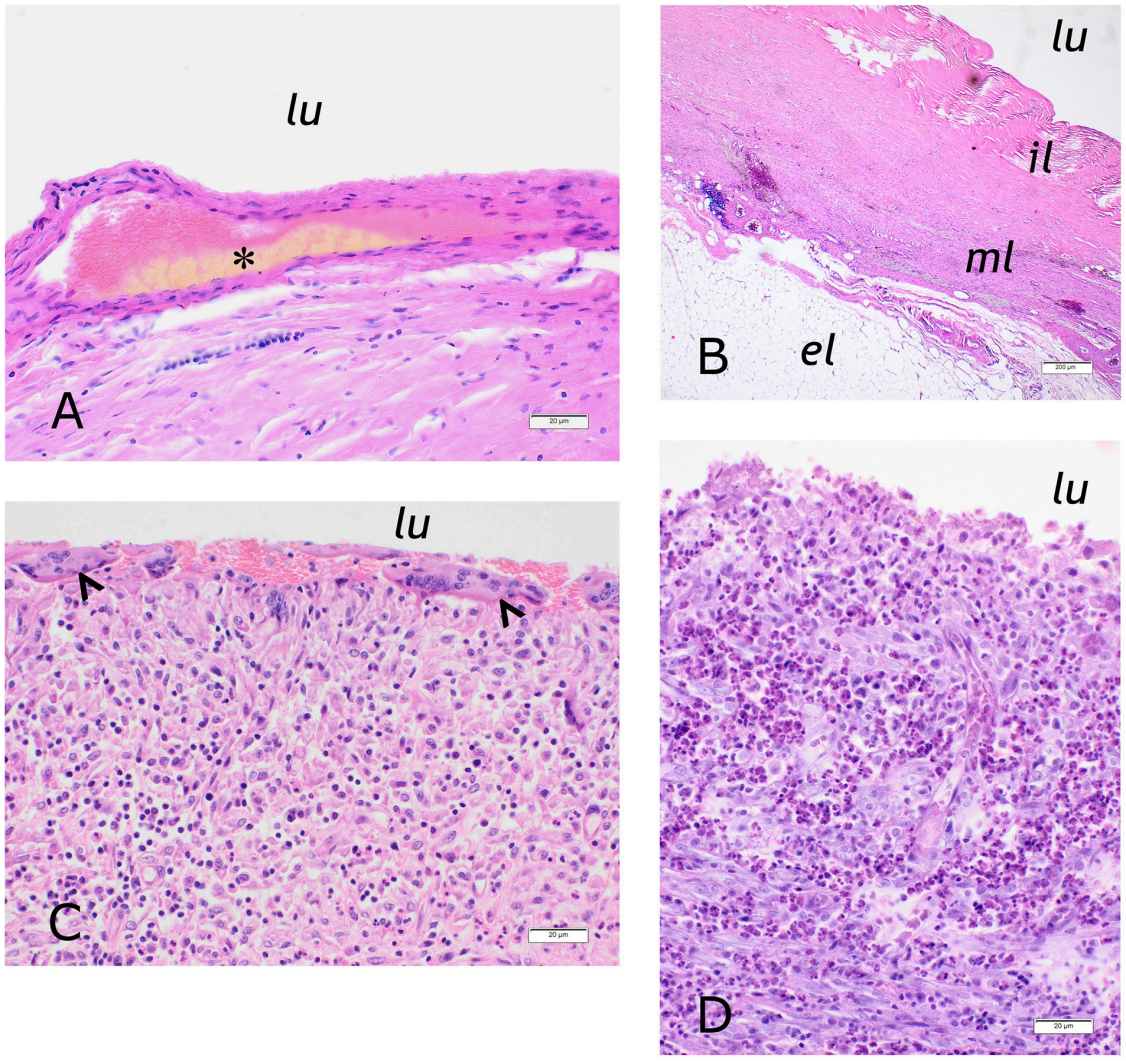
**(A)** Inner surface of the implant capsule of bear BD23, a 26-years old female which had carried the implant for 2 years. Aggregates of amorphous yellowish material (asterisk) were found close to the lumen (lu) and within a layer of sparsely cellular, laminar connective tissue (HE, bar = 20 μm). **(B)** Three-layered appearance of the implant capsule of bear W9403, a 23-years old female that had carried the implant for 3 years. The inner layer (il) consisted of partially necrotic dense and organized connective tissue. The middle layer (ml) consisted of laminar, sparsely cellular connective tissue and contained vessels and occasional hemorrhages, infiltrates of lymphocytes and aggregates of foamy, slightly greenish macrophages. The outer layer (ol) was composed of fat cells (HE, bar = 0,02 mm). **(C)** Foreign body reaction in the inner layer of the implant capsule of bear BD155, a 22-years old female that had carried the implant for 1 year. Multinucleated giant cells (arrowheads) lined the inner surface. Below were infiltrates of epitheloid and normal macrophages, proliferating vessels and some eosinophilic and neutrophilic granulocytes (HE, bar = 20 μm). **(D)** Inner layer of the implant capsule of case 2/bear W0104, a 12-years old female that had carried the implant for 3 years. The tissue was infiltrated by large numbers of eosinophilic granulocytes (HE, bar = 20 μm).

We took biopsies from the connective tissue capsule surrounding the implants from 33 bears. One individual was sampled twice. Of these, one had carried the implant for 9 years, two for 8 years, three for 7 years, three for 6 years, 11 for 5 years, four for 4 years, six for 3 years, two for 2 years, and two for 1 year (Figure 2B). Histological examination showed that the tissue was organized into three layers (Figure 3). The inner surface was sometimes covered by proliferating serosa, but in most cases, this layer was characterized by necrotic dense connective tissue organized in a regular and laminar pattern. Aggregates of yellowish, amorphous material were often located close to the surface. Areas of necrosis were found in most of the bears that had carried the implant for ≥4 years (20 of 24), whereas necrosis only was found in two of the six bears that had carried the implant for 3 years, and in none of the four that had carried it for < 3 years (Figure 2C). The inner layer was otherwise characterized by well-organized, collagen-rich and sparsely cellular connective tissue. Here, macrophages containing yellowish, greenish or brownish material were often found solitarily or in small aggregates. A significant inflammatory reaction was evident in a third of the biopsies, with the tissue characterized by granulomatous inflammation. The most severe cases contained numerous macrophages, epithelioid cells, multinucleated giant cells, neutrophilic granulocytes, fibroblasts, and proliferating vessels. In six of the cases, eosinophilic granulocytes constituted a significant proportion of the cellular infiltrate. The capsules from bears that had carried the implant for a long period were generally characterized by a milder inflammatory reaction than those that had carried it for a shorter period. However, we observed granulomatous inflammation in most of the cases, as only seven of the inspected capsules did not show any inflammatory cellular reaction. The middle layer of the capsule was often characterized by a well-organized, laminar connective tissue, but more active cases showed a more irregular organization with bundles and streams of highly cellular connective tissue and abundant vascularization. Prevalent findings were solitary clusters of large, foamy macrophages, often with a yellowish discoloration of the cytoplasm, and multiple, small aggregates of macrophages containing dark, brownish pigment granulae. Vascular proliferation, numerous hemorrhages and multifocal perivascular aggregates of lymphocytes were also prevalent findings. The third, outer, layer of the capsule most often consisted of adipose tissue, sometimes with mild perivascular infiltrates of lymphocytes, but often without any obvious inflammatory cellular response.

### Below we report details on seven selected individual bears

Capture, handling and treatment of bears included in Cases 1–7 below, were carried out in accordance with an established protocol (20).

### Case 1 (Figure 4)

A female bear (W8906), born in 1981 was implanted with a Telonics IMP/400/L/HP in April 1997. The implant was removed after 8 years in April 2005. The transmitter was encapsulated within the greater omentum, which was twined around its axis in a complete volvulus, so that the proximal part of the omentum formed a rope-like structure. We amputated the greater omentum involved in the twist. The capsule wall consisted of a 10–15 mm thick, grayish, and fleshy layer of connective tissue with rich vascular supply. The internal surface was irregular and yellowish, and the external serosa was hyperemic. There was a sticky, yellowish, hemorrhagic and odorless fluid between the capsule and the implant. The swab culture was negative. Histologically, the capsule wall consisted mainly of densely woven connective tissue without any obvious organization. Foci of necrotic fat were found in some areas and small hemorrhages, moderate perivascular infiltrates of inflammatory cells, mainly lymphocytes, and a small number of macrophages with dark pigments, were widely distributed. There were abundant proliferating vessels within the connective tissue. The surface of the implant had a yellowish discoloration. The inside of the plastic cylinder was covered with small, clear droplets of fluid. Both layers of the paper wrapping were moist and the batteries and other metal parts were corroded. This bear had produced four litters, with three cubs in each, in 1998, 2000, 2002, and 2004. It was shot legally in October 2005, but the hunter provided no information about the carcass.

**Figure 4:**
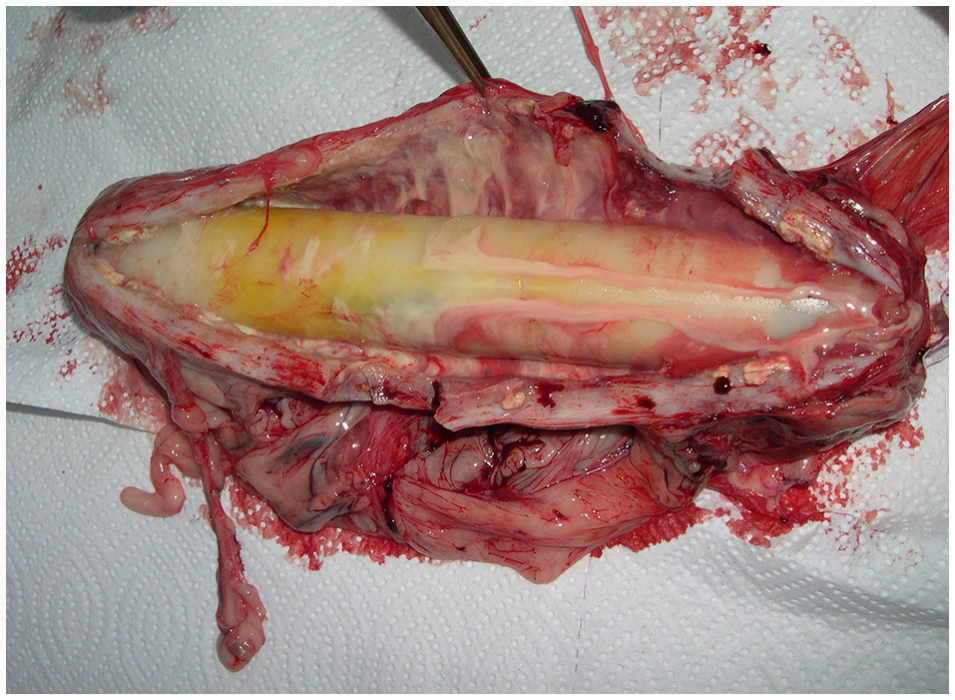
Implant (Telonics IMP/400/L/HP, 21.0 × 3.3 cm) and capsule from a 24-years old female bear (W8906) that had carried the device for 8 years.

### Case 2 (Figure 5)

A female bear (W0104), born in 2000 was implanted with a Telonics IMP/400/L in April 2001. The implant was removed after 5 years in April 2006. The transmitter was found in the greater omentum, surrounded by a 5 mm thick capsule. A round, cystic, abscess-like structure 5 cm in diameter was attached to one side. We amputated the capsule and abscess. Both the capsule and the cystic structure contained a yellow, thick, opaque, and odorless fluid without any recognizable distinct odor. Three culture swabs from the capsule and fluid were negative. The internal surface of the capsule wall was smooth and displayed areas of orange discoloring. The texture of the capsule was firm, but the external layer of the tissue consisted of fat. Histologically, the internal part of the capsule was composed of laminar, loosely woven, and mostly necrotic tissue, which was diffusely infiltrated with macrophages and some neutrophilic granulocytes. The middle part consisted of dense connective tissue without any particular organization and the external layer consisted of fat infiltrated with macrophages and neutrophilic granulocytes. The blood vessels of the capsule were surrounded by mild to moderate infiltrates of lymphocytes. The coating of the implant was intact, but slightly discolored. No other damage was observed in other parts of the implant. This bear was implanted again in April 2009, and had the implant replaced both in April 2012 and April 2015 (Telonics IMP/700 in all occasions). No pathological changes were observed during these surgeries. The bear had litters in 2005 (two cubs), 2008 (three cubs), 2011 (two cubs), and 2014 (three cubs) and was alive at den entry 2015.

**Figure 5:**
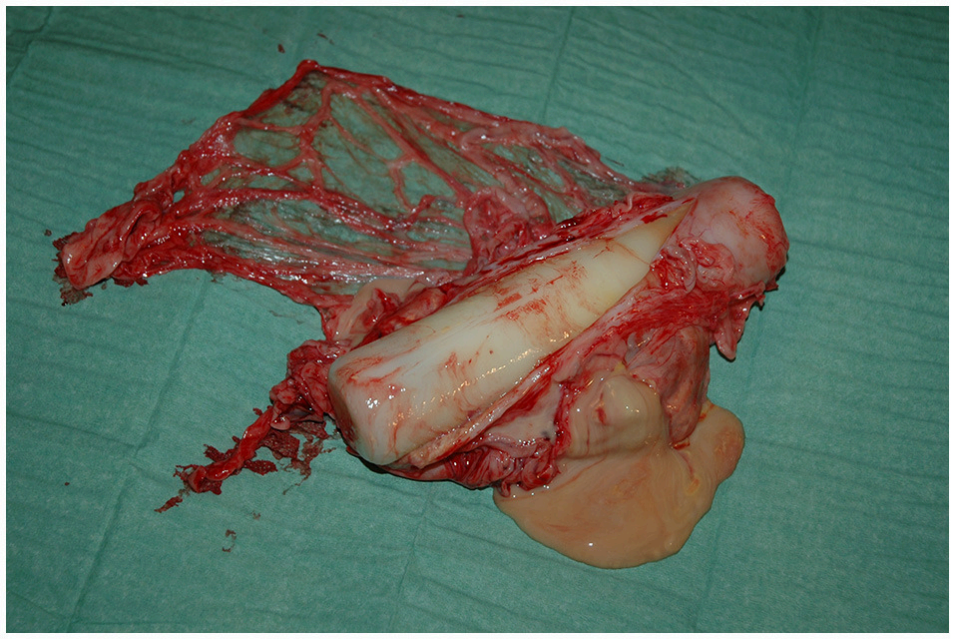
Implant (Telonics IMP/400/L, 15.2 × 3.3 cm) and capsule with abscess-like cyst from a 6-years female bear (W0104) that had carried the device for 5 years.

### Case 3 (Figure 6)

A female bear (W0010), born in 1999 was implanted with a Telonics IMP/400/L in April 2000. The transmitter was replaced after 6 years in April 2006. The implant was in the greater omentum, encapsulated by a 1 mm thick layer of tissue that did not require amputation to remove the transmitter. The tissue had irregular whitish surfaces and a texture resembling fat. Histologically, the tissue consisted of moderately vascularized, sparsely cellular adipose tissue with small, multifocal necrotic areas. The blood vessels within this tissue were surrounded by moderate amounts of connective tissue. On inspection, the wax coating of the implant had a “melted” appearance. One end cap was loose and the internal parts could not be removed without cracking the plastic shell cylinder. The paper wrappings appeared scorched and the batteries had short-circuited and were adherent to the inner surface of the plastic shell cylinder. This bear had produced litters with two cubs in 2005, 2007, and 2009. It was shot legally in September 2009, but the hunter provided no information about the carcass.

**Figure 6:**
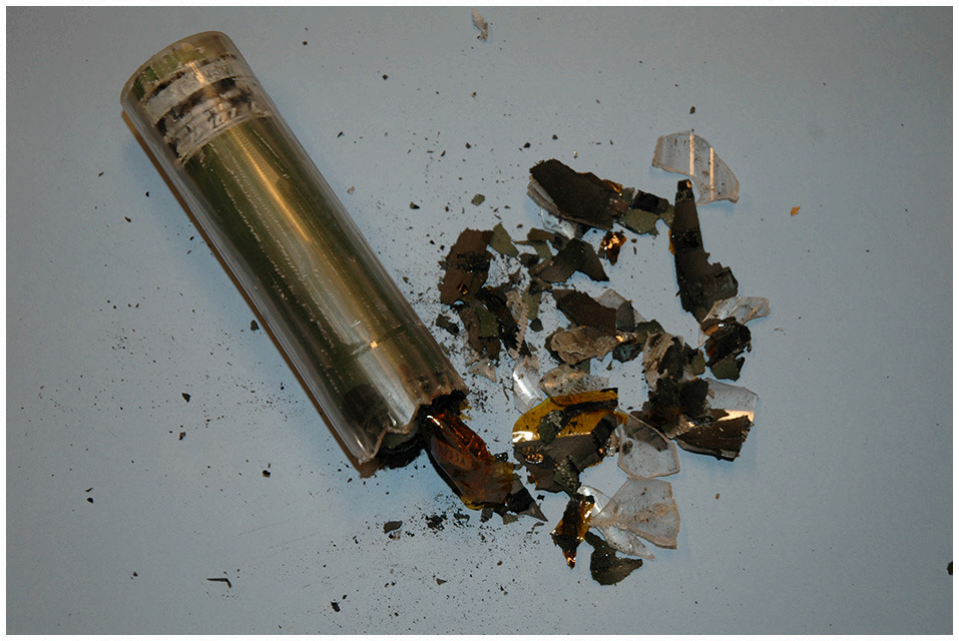
Implant (Telonics IMP/400/L, 15.2 × 3.3 cm) removed from a 7-years old female bear (W0010) that had carried the device for 6 years. The batteries had short-circuited, with subsequent over-heating of the device. The wax coating had a “melted” appearance and one end cap was detached. The batteries were adherent to the inside wall of the plastic cylinder, which had to be cracked to remove the internal parts of the device.

### Case 4 (Figure 7)

A female bear (BD124), born in 2001, was implanted with a Telonics IMP/400/L in May 2002. The transmitter was removed after 5 years in May 2007. When the peritoneal cavity was opened, the author who carried out the surgery (JA), could smell a “battery-like” odor and found that the implant was encapsulated in a 15-cm wide mass of connective tissue attached to the ventral abdominal wall cranially to the umbilicus. Parts of this tissue, including the core containing the transmitter, were removed and the abdominal cavity was closed. The tissue covering the implant was firmly attached to it and had to be removed with a scalpel. One end cap was detached. Closer inspection revealed that the implant clearly had over-heated. The outside of the plastic cylinder was partly covered by a soot-like material, and the inside smelled of battery. All internal parts of the transmitter appeared to be burned and were covered by the same soot. Both batteries had obviously been exposed to high temperatures and were partly open along the sides. The inside of the tissue that covered the implant was dark and irregular and had a “fried meat” appearance, whereas the outer surface was smooth and pale reddish. Histologically, the inner surface consisted of partly necrotic, finger-like projections of connective tissue. Next to this, the capsule wall consisted of connective tissue organized in a woven pattern. In the wall, there were large accumulations of lipid-like, brownish material in the tissue, pockets of macrophages filled with yellow/brown material, and foci of calcification and mild hemorrhages. The external layer of the capsule consisted of necrotic adipose tissue with spots of yellowish discoloration. The bear had its first litter (3 cubs) the year following implantation and was legally shot in May 2009 to avoid predation of semi-domesticated reindeer in a calving area. No pathological changes were noted at necropsy, but the report focused on the trauma from three rifle shots.

**Figure 7:**
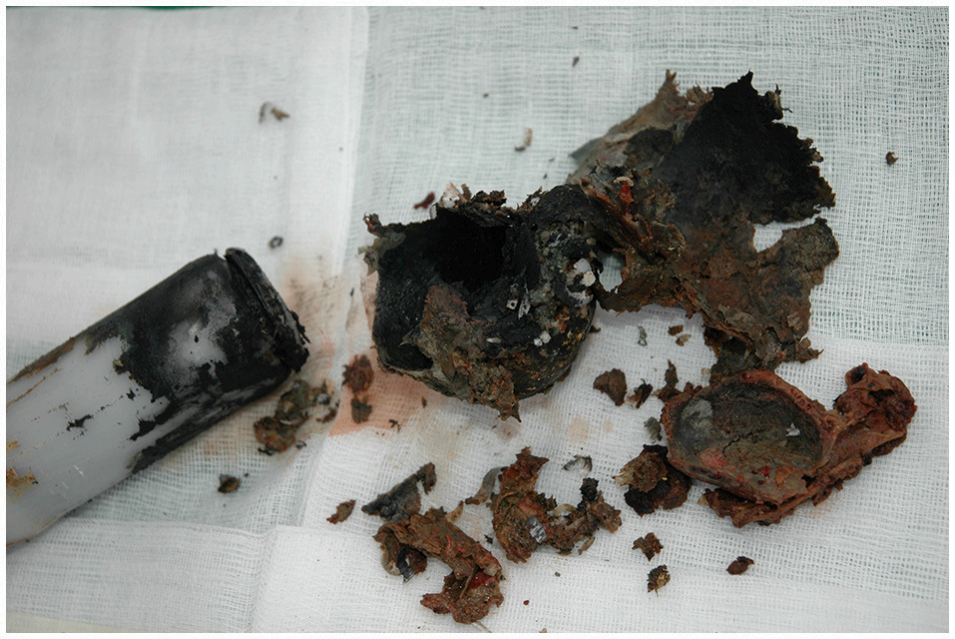
Implant (Telonics IMP/400/L, 15.2 × 3.3 cm) removed from a 6-years old female bear (BD124) that had carried the device for 5 years. The batteries had short-circuited, with subsequent over-heating of the device and leakage from the batteries. One end cap was detached and the outside of the implant was partly covered by a soot-like material. All internal components appeared to be burned and were covered by the same soot.

### Case 5 (Figure 8)

A female bear (W0020), born in 1999, was implanted with a Telonics IMP/400/L in April 2000. This bear went missing (no radio signal) in October 2002, but was found dead in September 2010, 10 years after surgery. At necropsy, the implant was found free-floating in the abdomen. One end cap was completely detached and a metal wire (antenna) had perforated the stomach. The cause of death was peritonitis with subsequent sepsis. The wax coating was melted at both ends of the implant. The plastic cylinder had multiple, longitudinal cracks and fell into multiple pieces when the wax was removed. All internal parts were literally burned, presumably due to short-circuiting of the batteries and subsequent over-heating.

**Figure 8:**
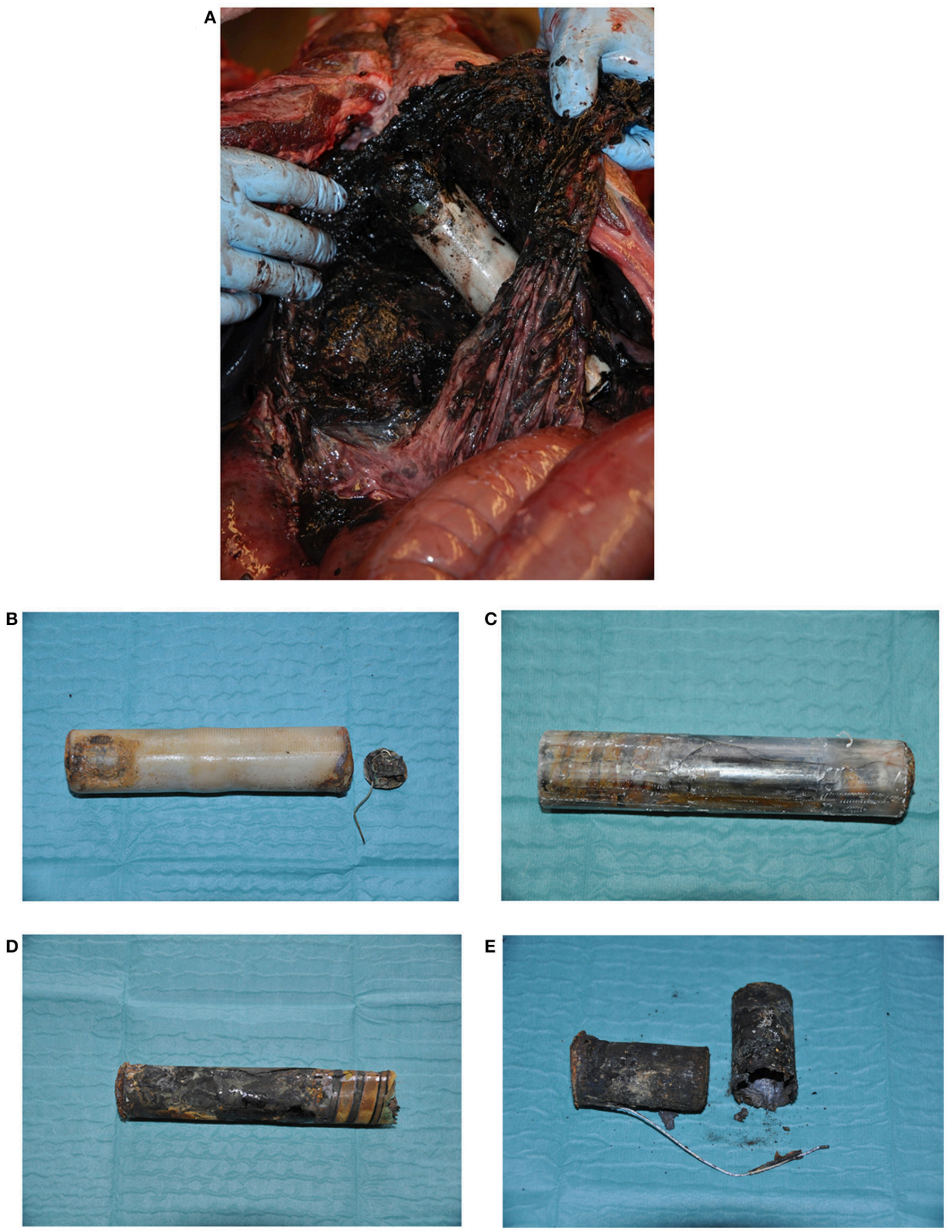
Implant (Telonics IMP400/L, 15.2 × 3.3 cm) from a female bear (W0020) found dead 10 years after implantation. Cause of death was peritonitis, with subsequent sepsis, due to perforation of the stomach by a metal wire (antenna) and leakage from the batteries that had short-circuited. **(A)** Implant *in situ* at necropsy. **(B)** Implant with detached end cap and metal wire. **(C)** Implant after removal of the wax coating, showing several longitudinal cracks in the plastic cylinder. **(D)** Outer paper wrapping, after removal of the plastic cylinder, showing signs of over-heating and scorching. **(E)** Batteries showing signs of over-heating.

### Case 6

A male bear (BD142), born in 2001 was implanted with a Telonics IMP/400/L in May 2002. This bear went missing (no radio signal) in September 2005, but was found dead in July 2015, 13 years after surgery. The cadaver was decomposed, but otherwise intact, except for a 10 × 15 cm opening into the abdominal cavity. The implant was located close to this opening. The bear's body condition was average and the gastrointestinal tract was empty. The pathologist concluded that death had occurred quickly. The implant was covered with a black material, one end cap was open, and the inside of the plastic cylinder contained abundant, black, and sticky material. The plastic cylinder was cracked and the batteries had short-circuited and were leaking. Although the necropsy report was not conclusive, it is likely that leakage from the batteries caused fatal peritonitis, with possible erosion of the abdominal wall.

### Case 7 (Figure 9)

A female bear (BD109), born in 1999 was implanted with a Telonics IMP/400/L in May 2000. The implant was removed after 5 years in May 2005. The transmitter was in the greater omentum, surrounded by a 2–3 mm thick capsule with a rich vascular supply. We amputated part of the omentum, which was in a full volvulus, with its root having the appearance of a twisted rope. The wall of the capsule had a smooth inner surface and an outer surface that was covered with adipose tissue. The middle layer consisted of fibrous tissue containing some gray to yellow tissue. Histologically, the inner layer consisted of necrotic, laminar, and loosely woven tissue over a zone of necrotic debris without any obvious organization. The middle layer consisted of laminar, densely woven connective tissue, and the external surface was covered by adipose tissue. Spots of calcification, small hemorrhages, and mild, diffuse infiltrates of neutrophilic granulocytes were present in the tissues. Some epithelioid macrophages with foamy cytoplasm were also seen, and there were multiple aggregates of yellow pigment within the capsule wall. Except for minor signs of wax wear at the ends, all parts of the implant were unremarkable. This bear was captured again in May 2008 to change the radio collar and in May 2011 to remove the radio collar. It produced two cubs in 2007 and 2010. No further information about its fate after 2011 is available.

**Figure 9:**
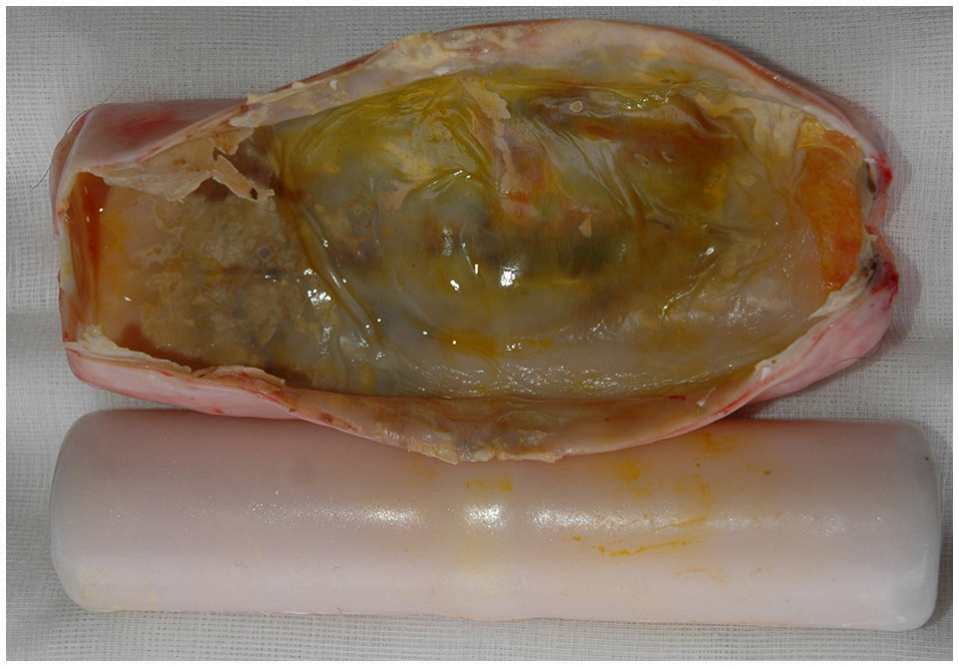
Implant (Telonics IMP/400/L, 15.2 × 3.3 cm) and capsule from a 6-years old female bear (BD109) that had carried the device for 5 years.

## Discussion

We documented that Telonics intraperitoneal VHF radio transmitters had a high rate of serious technical failures, including an ineffective moisture barrier that caused corrosion of metal parts. We also documented that implants can over-heat and disintegrate, due to short-circuiting of the batteries, causing serious tissue trauma in one bear after 5 years and killing two other individuals after 10 and 13 years.

Telonics (21) has stated that the wax coating was stable at physiological temperature of 5–50°C. However, we observed several cases of changes in the coating consistent with partial melting. The normal deep body temperatures of brown bears are 32–34°C during hibernation and 37–38°C during mid-summer (22). The highest core body temperature we have recorded in this species, using abdominal temperature loggers, is 42.0°C. We doubt that bears can survive deep body temperatures exceeding 43–44°C and 50°C is definitely not survivable. Consequently, the wax coating is either not stable up to 50°C, as stated by the manufacturer, or the device can generate internal heat that is sufficient to melt the wax.

Our finding of a mild to moderate granulomatous inflammation in the capsule surrounding the implant in nearly half of the instrumented bears, shows that the wax coating is not physiologically compatible. This is supported by the finding in some cases of macrophages containing granulae with yellowish pigment consistent with wax. We interpret this as a mild to moderate foreign body reaction to the coating (23). Furthermore, entrapment of the implant in the greater omentum caused a volvulus in several cases. In humans, volvulus of the greater omentum is associated with acute abdominal pain (24).

Tissue reactions to the implant appear to have started when the device became trapped in the greater omentum, i.e., encapsulation can occur in less than a year or may never happen. Four out of eight implants removed after 1 year were encapsulated. Of the 66 implants removed after 1–3 years, 30 were encapsulated and seven required amputation. On the other extreme, nine implants were found free-floating after 6–9 years.

Telonics (25) does not provide information about the chemical composition of the wax coating used on the implants. In 1983, Telonics (21) stated that “After extensive *in vivo* testing of many specialized coatings and formulations, a particular combination of physiological embedding wax and resin was determined to meet the specialized criteria for totally encapsulated telemetry units. The resultant coating is an effective moisture barrier to saline solutions, elicits little or no tissue reaction, and is stable at physiological temperatures (5–50°C).” The current product information (25) is that implants “have a dual water barrier, a sealed polycarbonate tube coated in wax; which completely encloses the transmitter electronics, power supply and the transmitting antenna. This design makes the implant less subject to mechanical damage and reduces the chance for moisture penetration over the life of the transmitter. This approach represents the best and most reliable packaging available for implants.” The company also states that “implants are well-tolerated” (25). There is, however, no scientific evidence to support any of these claims and the manufacturer does not provide any advice on whether or not the implant should be removed from an instrumented animal. We found corrosion of batteries and other metal parts after only 3 years in eight implants and damage of the batteries, consistent with short-circuiting in two implants after 5 years. We also found that nearly half of the implants were encapsulated, with necrotic and inflammatory tissue reactions. This shows that the wax coating of the implant is neither an effective moisture barrier nor biocompatible.

We are unaware of any recommendations in the published literature regarding whether an implanted radio transmitter should be removed or not. The Canadian Council on Animal Care (26) stated that external transmitters ideally should be removed once an experiment or study is completed. These guidelines also cover implantable transmitters, but nothing is said about the removal of such devices. In 2018, Telonics is marketing a model (IMP/700/2) with an operational life of 10 years (25). Instrumentation is thus clearly meant to be long-term and potentially lifelong in most species.

In 1997, the SBBRP started using Telonics implants as an alternative to collars in yearling bears and later as a back-up VHF transmitter in adults equipped with GPS-collars. Based on observations during this study, our standard procedure during the past decade has been to change or remove the implants after 3–5 years, depending on the reproductive cycle of females and other considerations. Due to concerns over the poor technical quality of the implants and adverse reactions to the wax coating, the SBBRP has decided to stop using Telonics implants and all such devices carried by bears that still can be radio-tracked, will be removed.

## Conclusions

In our opinion, these intraperitoneal radio transmitters should not be used in brown bears.

We have documented how a lack of attention to biological compatibility and technical stability of implanted devices can have drastic welfare implications for study animals. We recommend that standards similar to those used in human medicine be adapted for the development and use of intraperitoneal radio transmitters in wildlife.

## Data availability statement

The raw data supporting the conclusions of this manuscript will be made available by the authors, without undue reservation, to any qualified researcher.

## Author contributions

JA: study concept and design; JA, BY, KM, JM, SB, PS, AE, and JS: acquisition and interpretation of data; JA, KM, SB, PS, and AE: capture and handling of bears; JA, KM, and AE: surgeries and sampling; BY and KM: histopathology; JM: necropsy; JA and SB: technical inspection of transmitters; JA and BY: drafting of manuscript; JA, BY, KM, JM, SB, PS, AE, and JS: critical revision of manuscript and approval of submitted version.

### Conflict of interest statement

The authors declare that the research was conducted in the absence of any commercial or financial relationships that could be construed as a potential conflict of interest.
